# Failure of remission induction by glucocorticoids alone or in combination with immunosuppressive agents in IgG4-related disease: a prospective study of 215 patients

**DOI:** 10.1186/s13075-018-1567-2

**Published:** 2018-04-10

**Authors:** Liwen Wang, Panpan Zhang, Mu Wang, Ruie Feng, Yamin Lai, Linyi Peng, Yunyun Fei, Xuan Zhang, Yan Zhao, Xiaofeng Zeng, Fengchun Zhang, Wen Zhang

**Affiliations:** 1 Department of Rheumatology, Peking Union Medical College Hospital (West Campus), Chinese Academy of Medical Science & Peking Union Medical College, Key Laboratory of Rheumatology and Clinical Immunology, Ministry of Education, No.41 Da Mu Cang, Western District, Beijing, 100032 People’s Republic of China; 2 Tsinghua University School of Medicine, Beijing, 100086 China; 3 Department of Stomatology, Peking Union Medical College Hospital, Beijing, China; 4 Department of Pathology, Peking Union Medical College Hospital, Beijing, China; 5 Department of Gastroenterology, Peking Union Medical College Hospital, Beijing, China

**Keywords:** IgG4-related disease, Remission, Relapse, Glucocorticoids, Immunosuppressive agents

## Abstract

**Background:**

The aim of this study was to assess the outcomes of remission induction in patients with IgG4-related disease (IgG4-RD) in our cohort, and to investigate the characteristics, prognosis, and risk factors in the patients failed of remission induction.

**Methods:**

We prospectively enrolled 215 newly diagnosed patients with IgG4-RD, who were initially treated with glucocorticoid (GC) alone or in combination with immunosuppressive agents (IM), and had at least 6 months of follow up. The therapeutic goals of remission induction were defined as fulfilling each of the following after the 6-month remission induction stage: (1) ≥ 50% decline in the IgG4-RD responder index (RI); (2) GC tapered to maintenance dose; and (3) no relapse during GC tapering. The patients not achieving the therapeutic goals were considered to have failed of remission induction.

**Results:**

There were 26 patients in our cohort who failed of remission induction, including 16 (20.8%) on GC monotherapy, and 10 (7.2%) on combination therapy comprising GC and IM. The lacrimal gland and lung were most common sites of remission induction failure. Among the patients who relapsed during remission induction stage, 52.9% had secondary relapse during follow-up. Eosinophilia, higher baseline RI, more than five organs involved and dacryoadenitis were risk factors for remission induction failure with GC monotherapy, and the incidence of remission induction failure was 71.4% in the patients with more than three risk factors. After 6-month treatment, the patients who failed of remission induction had significantly higher erythrocyte sedimentation rate (ESR), C-reactive protein (CRP) and IgG4.

**Conclusion:**

In our cohort, 20.8% of patients failed of remission induction with GC monotherapy, while 7.2% of patients failed of remission induction with combination therapy comprising GC and IM.

**Electronic supplementary material:**

The online version of this article (10.1186/s13075-018-1567-2) contains supplementary material, which is available to authorized users.

## Background

IgG4-related disease (IgG4-RD) is an immune-mediated systemic fibro-inflammatory disease, which is characterized by enlargement of the involved organs, elevated serum concentrations of IgG4, and pathology findings of dense lymphoplasmacytic infiltration enriched in IgG4-positive plasma cells, storiform fibrosis, obliterative phlebitis and mild-to-moderate eosinophilia [1–4].

Glucocorticoid (GC) is the standard first-line agent for remission induction [5]. Patients with IgG4-RD typically respond well to initial GC treatment, with improvement of symptoms and signs, resolution of enlarged organs and decrease in serum IgG4 levels [6–10]. In addition, combination therapy comprising GC and immunosuppressive agents (IM) has also been shown effective, while the necessity of combination therapy from the beginning of treatment remains controversial [11–16].

Of note, there is a small minority of patients with less favorable response to remission induction, including those who have persistently active disease despite treatment, relapse during GC tapering or fail of GC tapering because of unstable disease [12, 17–20]. However, there are limited data on patients with less favorable response.

Here, we assessed the outcomes of remission induction  in 215 patients with IgG4-RD in our prospective cohort, who were treated with GC alone or in combination with IM, and investigated the characteristics, prognosis, and risk factors among patients who failed of remission induction.

## Methods

### Patient enrollment

In our prospective cohort of patients with IgG4-RD in the Peking Union Medical college hospital [9], registered on ClinicalTrials.gov (ID: NCT01670695), 444 patients were enrolled from 2012 to 2017. Among them, we included all the newly diagnosed patients with IgG4-RD, who were initially treated with GC alone or in combination with IM, and had at least 6 months of follow up (n = 215). All patients satisfied the 2011 comprehensive diagnostic criteria for definite, probable or possible IgG4-RD [21]. Patients with other rheumatic diseases, infectious diseases or malignancies were excluded. In addition, patients with conditions that could mimic IgG4-RD were excluded. The study protocol was approved by the Ethics Committee of Peking Union Medical College Hospital. All enrolled patients consented to attend this cohort study and signed written informed consent.

### Laboratory tests, imaging studies and histological examination

Complete blood count, urinalysis, liver and renal function tests, erythrocyte sedimentation rate (ESR), hyper-sensitivity C-reactive protein (CRP), serum immunoglobulin levels and IgG subclasses were tested. All patients underwent imaging examinations, including ultrasonography, computed tomography (CT), magnetic resonance imaging (MRI) or positron emission tomography/computed tomography (PET-CT). Tissue biopsies were obtained from 112 patients, and samples were analyzed using previously described pathology methods [22].

### Treatment regimens

All the patients were treated with prednisone at 0.5 ~ 1.0 mg/kg body weight/day (30 ~ 60 mg/day). The initial dose of prednisone was continued for 2 weeks to 1 month, then gradually tapered by 5 mg per 2 weeks to the maintenance dose of 5 ~ 10 mg/day. Patients treated with additional IM (n = 138) received cyclophosphamide (CTX) (n = 67), mycophenolate mofetil (MMF) (n = 47), methotrexate (MTX) (n = 12), azathioprine (AZA) (n = 6), tripterysium glycosides (T2) (n = 3), leflunomide (LEF) (n = 1), cyclosporine A (CyA) (n = 1) and CTX + LEF (n = 1).

### Assessment of disease activity and definition of relapse

Organ involvement was evaluated by medical history, physical examination, laboratory tests, imaging studies and tissue biopsies. Disease activity was assessed by the IgG4-RD responder index (RI) [23]. Follow-up visits were scheduled at months 1, 3 and 6, and once every 3 ~ 6 months afterwards. During follow up, laboratory tests and imaging studies were performed when necessary. The patients were considered as having a relapse when they had recurrence of symptoms and signs or worsening on imaging studies, with or without re-elevation of serum IgG4.

### Therapeutic goals of remission induction and assessment of treatment outcomes

**Fig. 1:**
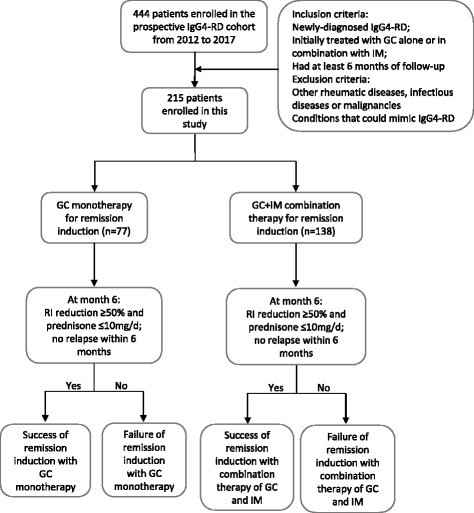
Flow scheme of patient enrollment, grouping according to treatment and assessment of treatment outcomes. IgG4-RD, IgG4-related disease; GC, glucocorticoid; IM, immunosuppressive agents

The initial 6 months was defined as the remission-induction stage, and the therapeutic goals of remission induction were defined as fulfilling each of the following after 6-month treatment: (1) ≥ 50% decline in the IgG4-RD RI; (2) GC tapered to maintenance dose (prednisone ≤10 mg/day); and (3) no relapse during GC tapering (within 6 months). The patients who reached the therapeutic goals were considered to have successful remission induction, while remission induction was considered to have failed in the other patients. In addition, the patients were grouped according to the treatment regimens they received during the remission-induction stage. The flow scheme of patient enrollment, grouping and assessment of treatment outcomes is shown in Fig. 1.

### Statistical analysis

Statistical analysis was performed using IBM SPSS (version 23) and R studio (version 3.4.0). Analysis by the Kolmogorov-Smirnov test revealed that data on age, disease duration, RI, number of organs involved, eosinophils, ESR, CRP, IgG, IgG4, IgG4/IgG and IgE were not normally distributed. All the continuous non-normally distributed data were presented as median (first quartile, third quartile) and analyzed by non-parametric test. Categorical variables were assessed by the chi-square test or Fisher’s exact test. Univariate logistic regression was performed to investigate the risk factors for remission induction failure. Multivariate logistic regression was limited by the number of outcome events and the co-linearity among candidate risk factors. A *p* value < 0.05 was considered statistically significant.

## Results

### Patient demographic and baseline features

Demographic and baseline features of 215 patients with IgG4-RD enrolled in the study are presented in Table 1. There were 148 male and 67 female patients (male to female 2.2:1). Median age was 54 years (range 9 ~ 83 years). Median disease duration at the time of diagnosis was 12 months (range 10 days ~ 20 years). The most common clinical manifestations included Mikulicz’s disease, autoimmune pancreatitis, sclerosing cholangitis, retroperitoneal fibrosis, lung disease, sinusitis and lymphadenopathy.

**Table 1:** Baseline characteristics of patients with IgG4-RD (n = 215)

Variable	Value
Sex (male:female)	2.2:1
Age (years)	54 (46, 62)
Diagnosis, *n* (%)	
Definite	102 (47.4%)
Probable	10 (4.7%)
Possible	103 (47.9%)
Disease duration (months)	12 (4, 36)
IgG4-RD RI	14 (10, 18)
Allergy history, *n* (%)	115 (53.5%)
Number of organs involved, *n* (%)	
1 ~ 2	78 (36.3%)
3 ~ 4	101 (47.0%)
≥ 5	36 (16.7%)
Organ involvement, *n* (%)	
Mikulicz’s disease	138 (64.2%)
Dacryoadenitis	95 (44.2%)
Sialoadenitis	124 (57.7%)
Autoimmune pancreatitis	91 (42.3%)
Sclerosing cholangitis	58 (27.0%)
Retroperitoneal fibrosis	50 (23.3%)
Lung disease	62 (28.8%)
Kidney involvement	19 (8.8%)
Lymphadenopathy	139 (64.7%)
Sinusitis	62 (28.8%)
Prostatitis	30 (14.0%)
Skin involvement	12 (5.6%)
Aortitis/periaortitis	12 (5.6%)
Ophthalmic disease (except for dacryoadenitis)	7 (3.3%)
Mediastinal fibrosis	9 (4.2%)
Hepatopathy	6 (2.8%)
Thyroiditis	2 (0.9%)
Central nervous system involvement	2 (0.9%)
Laboratory tests at baseline	
Eosinophils (%)	3.2 (1.6, 6.3)
ESR (mm/h)	25 (10, 59.5)
CRP (mg/L)	3.78 (1.15, 10.81)
IgG (g/L)	18.87 (15.13, 24.53)
IgG4 (mg/L)	8960 (3500, 18,600)
IgE (kU/L)	362.5 (132.5, 774)

All the continuous non-normally distributed data are presented as median (first quartile, third quartile)

*IgG4-RD* IgG4-related disease, *RI* responder index, *ESR* erythrocyte sedimentation rate, *CRP* hyper-sensitivity C-reactive protein

### Response to treatment

**Fig. 2:**
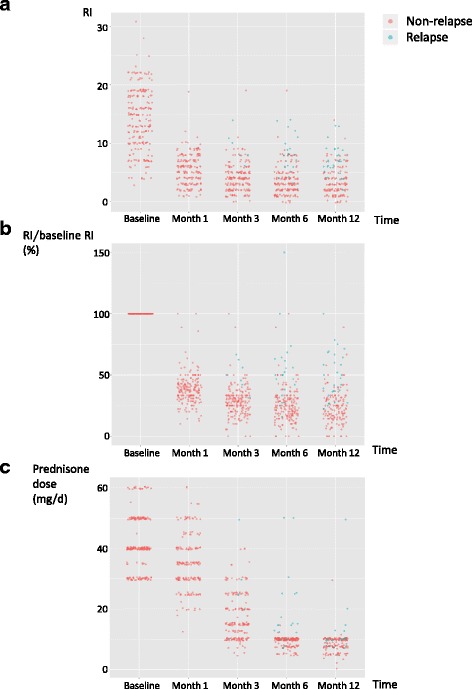
Follow-up data on 215 patients enrolled in the study. **a** Responder index (RI) at each visit. **b** RI at each visit in proportion to baseline RI. **c** Glucocorticoid (GC) doses at each visit. For the patients treated with GC other than prednisone, GC doses were adjusted to the equivalent prednisone dose

The median time of follow up was 22 months (range 6 ~ 60 months). The disease activity and GC dose at each visit are presented in Fig. 2. As shown in Fig. 2a and b, IgG4-RD RI decreased over time, especially during the first 3 months of treatment. In response to remission induction therapy, 189 (87.9%) patients reached remission without relapse, while 23 (10.7%) patients relapsed during GC tapering and 3 (1.4%) patients had persistently active disease despite 6-month treatment.

### Patients who failed of remission induction

As shown in Table 2, according to our definition, there were 26 (12.1%) patients in total who failed of remission induction, including 3 patients with decline in the IgG4-RD RI <50%, 23 patients who relapsed within 6 months and 17 patients who failed to have GC tapered to maintenance dose after 6 months. There was overlap between the patients who failed to have tapering of GC and patients with decline in the IgG4-RD RI < 50% or those who relapsed within 6 months. Significantly more patients failed of remission induction with GC monotherapy than with combination therapy (16/77, 20.8% vs 10/138, 7.2%, *p* = 0.008). Considering the heterogeneity of diagnostic status, we grouped the patients according to definite, probable and possible IgG4-RD, and found that the treatment outcomes were comparable in patients with different diagnostic status (Additional file 1). On the other hand, there was no significant difference in treatment-related side effects in patients treated with GC monotherapy or GC + IM combination therapy (Additional file 2).

**Table 2:** Baseline characteristics and outcomes of patients with IgG4-RD, grouped according to treatment

	GC monotherapy (n = 77)	Combination therapy comprising GC and IM (n = 138)	*p* value
Sex (male:female)	2.08:1	2.29:1	0.761
Age (years)	54 (45, 61)	54 (48, 62)	0.723
Disease duration (months)	12 (4, 24)	12 (4, 36)	0.649
IgG4-RD RI	13 (10, 18)	15 (10, 18)	0.685
Allergy history	42 (54.5%)	73 (53.7%)	1
Number of organs involved			0.369
1 ~ 2	32 (41.6%)	46 (33.3%)	
3 ~ 4	35 (45.5%)	66 (47.8%)	
≥ 5	10 (13.0%)	26 (18.8%)	
Organ involvement			
Mikulicz’s disease	51 (66.2%)	87 (63.0%)	0.659
Dacryoadenitis	35 (45.5%)	60 (43.5%)	0.886
Sialoadenitis	46 (59.7%)	78 (56.5%)	0.668
Autoimmune pancreatitis	34 (44.2%)	57 (41.3%)	0.774
Sclerosing cholangitis	22 (28.6%)	36 (26.1%)	0.749
Retroperitoneal fibrosis	10 (13.0%)	40 (29.0%)	0.007
Lung disease	17 (22.1%)	45 (32.6%)	0.118
Sinusitis	25 (32.5%)	37 (26.8%)	0.433
Lymphadenopathy	53 (68.8%)	86 (62.3%)	0.374
Laboratory tests at baseline			
Eosinophils (%)	3.3 (1.7, 6.2)	3.2 (1.4, 6.4)	0.773
ESR (mm/h)	25 (9, 53)	25 (10, 65)	0.790
CRP (mg/L)	2.06 (0.79, 11.24)	4.01 (1.29, 10.32)	0.297
IgG (g/L)	18.83 (14.24, 25.54)	18.9 (15.2, 23.68)	0.747
IgG4 (mg/L)	10,200 (3590, 18,800)	8955 (3480, 18,075)	0.554
IgE (kU/L)	510 (178, 881)	256 (119, 728)	0.118
Initial GC dose	40 (35, 50)	40 (40,50)	0.567
Outcomes at month 6			
RI reduction^a^ <50%	2 (2.6%)	1 (0.7%)	0.292
Relapse	14 (18.2%)	9 (6.5%)	0.011
Failure of GC tapering	8 (10.4%)	9 (6.5%)	0.429
Failure of remission induction	16 (20.8%)	10 (7.2%)	0.008

All the continuous non-normally distributed data were presented as median (first quartile, third quartile)

*IgG4-RD* IgG4-related disease, *RI* responder index, *ESR* erythrocyte sedimentation rate, *CRP* hyper-sensitivity C-reactive protein, *GC* glucocorticoid, *IM* immunosuppressive agents

^a^Here we considered the maximum reduction during follow up, so that re-elevation due to relapse was not taken into account

### The organs that failed of remission induction

**Fig. 3:**
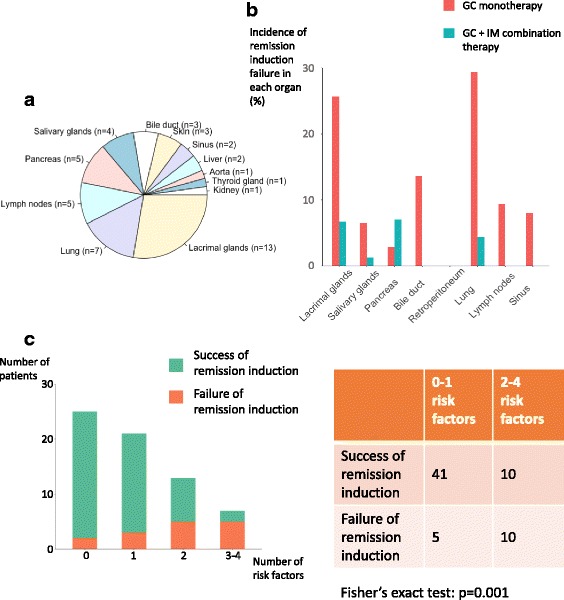
Organs that failed of remission induction. **a** Distribution of the organs that failed of remission induction. **b** Incidence of remission induction failure in each organ. The incidence was calculated as number of remission induction failures occurred in each organ/total number of patients with that particular organ involvement. **c** Treatment outcomes in the patients treated with glucocorticoid (GC) monotherapy, grouped according to the number of risk factors they had. Risk factors included eosinophilia, baseline responder index (RI) in the highest quartile, ≥ 5 organs involved and dacryoadenitis. IM, immunosuppressive agents

Distribution of the organs that failed of remission induction, namely the organs in which persistently active disease or relapse occurred, are presented in Fig. 3a. The lacrimal gland and lung were the most common sites of remission induction failure. In the patients treated with GC monotherapy (n = 77), the highest incidence of remission induction failure occurred in the lung, lacrimal gland and bile duct, accounting for 29.4% (5/17), 25.7% (9/35) and 13.6% (3/22), while in the patients treated with combination therapy (n = 138), the organs with the highest incidence of remission induction failure included the pancreas (7.0%, 4/57) and lacrimal gland (6.7%, 4/60) (Fig. 3b).

### Clinical features, treatments and outcomes in the patients who failed of remission induction

The clinical features, treatments and outcomes in the patients with persistently active disease or who relapsed during GC tapering are summarized individually in Additional files 3 and 4. Among the 23 patients who relapsed during GC tapering, 6 patients (26.1%) were treated with an increased dose of GC in combination with additional or alternative IM, 12 patients (52.2%) were treated with additional or alternative IM, 2 patients (8.7%) were treated with an increased dose of GC and 3 patients (13.0%) were treated with methylprednisolone (MP) pulse therapy, with or without adjustment of IM. Follow-up data after re-treatment were available in 21 patients, among whom 17 patients (81.0%) reached remission and 9 patients (52.9%) had another relapse after remission.

### Risk factors for remission induction failure

We performed logistic regression analysis to identify baseline risk factors for failure of remission induction (Table 3). Univariate logistic regression analysis revealed that in the patients treated with GC monotherapy, eosinophilia (OR = 7.27, 95% CI 2.05–25.74), higher baseline RI (highest quartile, OR = 8.44, 95% CI 1.48–48.14), more organs involved (≥ 5 organs, OR = 9.67, 95% CI 1.74–53.84) and dacryoadenitis (OR = 3.39, 95% CI 1.05–10.99) were associated with increased risk of remission induction failure. In addition, higher baseline IgG4 could be a potential risk factor, although it was not statistically significant (IgG4 >20 × upper limit of normal (ULN), OR = 4.5, 95% CI 0.89–22.67). In contrast, no significant association was identified between baseline characteristics and the risk of remission induction failure in the patients treated with combination therapy. Notably, the initial GC dose was not associated with the risk of remission induction failure in either group (OR = 1.03, 95% CI 0.97–1.1 and OR = 0.97, 95% CI 0.9–1.05), neither was the duration of the initial dose (GC tapered before week 4, OR = 0.75, 95% CI 0.2–2.83 and OR = 0.41, 95% CI 0.08–2.06). Multivariate logistic regression analysis was limited by the number of outcome events and the co-linearity among all the candidate risk factors.

**Table 3:** Univariate logistic regression analysis of risk factors for remission induction failure

	GC monotherapy		GC + IM combination therapy	
	OR (95% CI)	*p* value	OR (95% CI)	*p* value
Male sex	1.07 (0.33, 3.51)	0.907	4.24 (0.52, 34.6)	0.177
Age (continuous)	0.97 (0.93, 1.01)	0.103	1 (0.95, 1.05)	0.901
Disease duration (≥12 months)	1.02 (0.34, 3.1)	0.971	0.39 (0.1, 1.58)	0.186
Allergy history	1.51 (0.49, 4.68)	0.474	2.12 (0.52, 8.58)	0.291
Eosinophilia	7.27 (2.05, 25.74)	0.002	1.46 (0.39, 5.48)	0.576
Elevation of ESR	1.13 (0.33, 3.88)	0.847	1.03 (0.24, 4.54)	0.966
Elevation of CRP	1.65 (0.4, 6.86)	0.493	1.22 (0.28, 5.39)	0.791
IgG				
First quartile (median 12.78 g/L)	Ref	Ref	Ref	Ref
Second quartile (median 17.25 g/L)	0.50 (0.08, 3.15)	0.461	0.94 (0.12, 7.08)	0.949
Third quartile (median 21.30 g/L)	1.13 (0.21, 6.14)	0.892	1.76 (0.3, 10.31)	0.532
Fourth quartile (median 31.67 g/L)	1.41 (0.33, 5.98)	0.644	1.12 (0.15, 8.49)	0.916
IgG4				
IgG4 ≤3 × ULN	Ref	Ref	Ref	Ref
3 × ULN <IgG4 ≤10 × ULN	1 (0.2, 5.04)	1	Ref^a^	Ref^a^
10 × ULN <IgG4 ≤20 × ULN	1.64 (0.28, 9.58)	0.585	1.46 (0.35, 6.05)	0.604
IgG4 >20 × ULN	4.5 (0.89, 22.67)	0.068	NA^b^	NA^b^
IgE				
First quartile (median 75.5 kU/L)	Ref	Ref	Ref	Ref
Second quartile (median 203 kU/L)	1.45 (0.11, 18.96)	0.775	4.5 (0.44, 46.38)	0.206
Third quartile (median 529 kU/L)	1.07 (0.08, 13.65)	0.96	1.67 (0.1, 28.32)	0.724
Fourth quartile (median 1403 kU/L)	3.64 (0.35, 37.46)	0.278	3.16 (0.27, 37.27)	0.361
RI				
First quartile (median 7)	Ref	Ref	Ref	Ref
Second quartile (median 13)	0.95 (0.12, 7.44)	0.961	1.82 (0.24, 13.81)	0.563
Third quartile (median 16)	2.92 (0.47, 18.37)	0.253	1.08 (0.14, 8.07)	0.939
Fourth quartile (median 21)	8.44 (1.48, 48.14)	0.016	2.76 (0.47, 16.09)	0.259
Number of organs involved				
1 ~ 2	Ref	Ref	Ref	Ref
3 ~ 4	2.86 (0.69, 11.93)	0.148	0.92 (0.2, 4.34)	0.921
≥ 5	9.67 (1.74, 53.84)	0.01	1.87 (0.35, 10.02)	0.465
Organ involvement				
Mikulicz’s disease	4.54 (0.95, 21.78)	0.059	0.56 (0.15, 2.04)	0.38
Dacryoadenitis	3.39 (1.05, 10.99)	0.042	1.33 (0.37, 4.81)	0.667
Sialoadenitis	1.63 (0.51, 5.28)	0.412	0.75 (0.21, 2.73)	0.667
Autoimmune pancreatitis	1.35 (0.45, 4.06)	0.598	0.94 (0.25, 3.51)	0.931
Sclerosing cholangitis	0.8 (0.23, 2.8)	0.723	2 (0.53, 7.54)	0.306
Retroperitoneal fibrosis	NA^b^	NA^b^	0.25 (0.03, 2.07)	0.2
Lung disease	2.73 (0.82, 9.09)	0.103	1.41 (0.38, 5.29)	0.606
Initial GC dose	1.03 (0.97, 1.1)	0.271	0.97 (0.9, 1.05)	0.441
GC tapered before week 4	0.75 (0.2, 2.83)	0.671	0.41 (0.08, 2.06)	0.279

*Abbreviations: GC* glucocorticoid, *IM* immunosuppressive agents, *RI* responder index, *ESR* erythrocyte sedimentation rate, *CRP* hyper-sensitivity C-reactive protein, *OR* odds ratio, *Ref* reference, *ULN* upper limit of normal, *NA* not applicable

^a^The first two groups were combined as 0% remission induction failure occurred in the patients with IgG4 ≤3 × ULN

^b^Not applicable as 0% remission induction failure occurred in certain groups

To further investigate the impact of baseline risk factors on the incidence of remission induction failure, the patients treated with GC monotherapy were grouped according to the number of risk factors at baseline. As shown in Fig. 3c, the incidence of remission induction failure increased dramatically in the patients with more risk factors (8%, 14.3%, 38.5% and 71.4% when having 0, 1, 2 and 3–4 risk factors, respectively; *p* = 0.001 when grouping the number of risk factors into categories of 0–1 and 2–4).

### Comparison of serological features at month 6 in the patients who succeeded or failed of remission induction

**Fig. 4:**
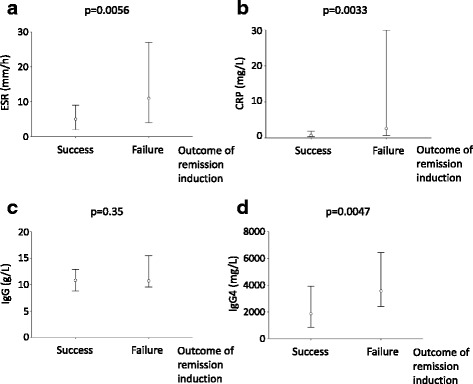
Serological features at month 6 in patients who succeeded or failed of remission induction. Levels of serum erythrocyte sedimentation rate (ESR) (**a**), hyper-sensitivity C-reactive protein (CRP) (**b**), IgG (**c**) and IgG4 (**d**) at month 6 were compared between the patients who succeeded or failed of remission induction. Data represent the median (first quartile, third quartile) of each parameter

To further investigate the characteristics of the patients who failed of remission induction, we compared the serological features after the remission-induction stage (at month 6) in the patients with success or failure of remission induction (Fig. 4). Interestingly, the patients who failed of remission induction had significantly higher ESR (mm/h) (11 (4, 27) vs 5 (2, 9), *p* = 0.0056), CRP (mg/L) (2.60 (0.70, 29.94) vs 0.73 (0.36, 1.83), *p* = 0.0033) and IgG4 (mg/L) (3570 (2413, 6443) vs 1870 (859, 3928), *p* = 0.0047), whereas IgG was comparable between these two groups.

## Discussion

In this article, we assessed the outcome of remission induction in 215 patients with IgG4-RD, who were treated with GC alone or in combination with IM, and focused on the subpopulation of patients with less favorable response to treatment.

Treat-to-target approaches, in which treatment regimens are escalated or adjusted until a specific target is reached, have been applied as routine management of patients with a variety of chronic diseases, and have been demonstrated to improve treatment outcomes significantly [24–28]. However, the therapeutic goals in IgG4-RD have not been clearly defined. It has been reported that in patients with IgG4-RD, persistently active disease could lead to fibrosis and irreversible organ damage [1, 2, 29]. Therefore, early intervention is critical, and it is also important to clarify the therapeutic goals of remission induction and confirm that the patients are receiving adequate treatment. Here, based on our experience, we defined the therapeutic goals of remission induction as fulfilling each of the following after 6-month treatment: (1) ≥ 50% decline in the IgG4-RD RI; (2) GC tapered to maintenance dose (prednisone ≤ 10 mg/day); and (3) no relapse during GC tapering (within 6 months). The patients not fulfilling the treatment goals were considered as failure of remission induction. We are aware of the possibility that the serum IgG4 level might not be lowered even if the treatment was effective, resulting in unsatisfactory decline in the IgG4-RD RI, and therefore some patients even in clinical remission might be classified as remission induction failure. However, in our cohort, only three patients failed of remission induction based on this criterion, and all three presented with persistently active disease in the affected organs, accompanied by failure of GC tapering to maintenance dose. Further validation is required for the treatment goals proposed by us.

Most of the patients reached remission through 6-month treatment, while 26 patients (12.1%) failed of remission induction (16 (20.8%) with GC monotherapy, and 10 (7.2%) with combination therapy comprising GC and IM). The baseline features were almost comparable between the two groups (Table 2), while the incidence of remission induction failure in the patients treated with GC monotherapy was significantly higher (*p* = 0.008). On the other hand, the patients treated with combination therapy did not experience more side effects compared to those treated with GC monotherapy (Additional file 2). It remains controversial whether some patients require the combination of GC and IM from the beginning of treatment [5]. Our data, along with previously published data showing that the patients treated with GC + CTX had a significantly higher rate of complete remission and lower rate of relapse [11], support the necessity of combination therapy with GC and IM as the initial treatment in certain patients.

Failure of remission induction most commonly occurred in the lacrimal glands and lungs (Fig. 3a). In the patients treated with GC monotherapy, lungs, lacrimal glands and bile duct had the highest incidence of remission induction failure (29.4%, 25.7% and 13.6%, respectively), indicating that the patients with involvement of these organs need more intensive follow up (Fig. 3b). In the patients treated with a combination of GC and IM, the overall incidence of remission induction failure was low, with relatively higher incidence in the pancreas and lacrimal glands (Fig. 3b).

Among the 23 patients who relapsed during GC tapering, re-treatment either with more intensive GC therapy or with the addition of or change in IM was effective in most of the patients (17/21, 81.0%), which is consistent with data from other studies [6, 12, 30]. Of note, among the patients who relapsed, secondary relapse occurred in 52.9%, which is much higher than the overall relapse rate with initial therapy (21.4%), indicating that the patients who have relapsed are susceptible to another relapse, and they need more intensive follow up.

Of note, there are four patients in our cohort (patient number 3, 15, 16 and 24) (Additional files 3 and 4), who had persistently active disease refractory to multiple treatment regimens. Although unresponsiveness to various treatment regimens is rare in IgG4-RD, diagnosis of IgG4-RD in those patients was confirmed by biopsy, and conditions that could mimic IgG4-RD, including anti-neutrophil-cytoplasmic antibody (ANCA)-associated vasculitis, multicentric Castleman’s disease, Kimura disease, Rosai-Dorfman disease and cancer, etc., were excluded. In accordance with our data, Wallace et al. reported three patients with IgG4-RD unresponsive to treatment with GC and/or rituximab [18]. Sun et al. reported three cases of IgG4-related lung disease, that were either unresponsive to a routine dose of GC, or failed GC tapering because of recurrent disease [20]. Taken together, it is possible that a small minority of patients with IgG4-RD is refractory to treatment, and further study on the management of those patients is required.

In the patients treated with GC monotherapy, eosinophilia, higher baseline RI, more than five organs involved and dacryoadenitis were identified as risk factors for remission induction failure (Table 3). Consistent with our data, it has been reported that higher baseline RI and greater number of organs involved are associated with worse outcomes with GC therapy [11, 19]. Eosinophilia is a common clinical manifestation in patients with IgG4-RD [3, 4]. Interestingly, it has been proposed that eosinophilia is associated with the inherent characteristics of IgG4-RD, rather than underlying allergy [31]. Our previous study also demonstrated that peripheral eosinophils was associated with disease severity, indicated by the number of organs involved and serum IgG4 levels [32]. Here, we also found that eosinophilia, rather than allergy history, is associated with treatment outcome. The etiology of eosinophilia in patients with IgG4-RD and its role in pathogenesis requires further investigation. As for particular organ involvement, dacryoadenitis is a newly discovered risk factor for worse outcome. In contrast to our study, it has been reported that in the patients with autoimmune pancreatitis, the presence of sclerosing cholangitis is a risk factor for relapse [6]. The discrepancy could be caused by the difference in disease subtypes and observation endpoints (failure of remission induction vs relapse).

When the patients treated with GC monotherapy were grouped according to the number of risk factors at baseline, we identified a trend towards higher incidence of remission induction failure in the patients with more risk factors (Fig. 3c). Of note, in the patients with more than three risk factors, the incidence of remission induction failure was as high as 71.4%. As aforementioned, remission induction failure was more common in the patients treated with GC monotherapy. Taken together, our data suggest the necessity of combination therapy with GC and IM for the patients with more than three risk factors.

In addition, after the remission-induction stage, serum ESR, CRP and IgG4 was significantly higher in the patients who failed of remission induction (Fig. 4), indicating the usefulness of these serological parameters to assess response to treatment. Consistent with our data, it has been reported that in patients with autoimmune pancreatitis, persistent elevation of IgG4 level after treatment is associated with persistent abnormalities of the pancreas and bile duct and more frequent relapse [7]. It has been proposed that the quantitation of circulating plasmablasts is better for monitoring disease activity than IgG4 level [18, 33]. However, considering the clinical availability, it is still valid to monitor serum IgG4 during follow up.

Our study also has some limitations. The biopsy-proven diagnosis rate was 52.1%, and the remaining patients were diagnosed based on clinical manifestation and serum IgG4 levels. However, all the patients without biopsy-proven diagnosis presented with typical features of IgG4-RD, conditions that could mimic IgG4-RD were excluded and the treatment outcomes were comparable in the patients with different diagnostic status (Additional file 1). In addition, the therapy regimens are varied between patients, including the initial doses of GC, the duration of initial dose and the type of IM. However, in logistic regression analysis there was no association between the risk of remission induction failure and either the initial GC dose or duration of the initial dose (Table 3). In addition, previous research from our group found no significant difference in the efficacy of high and medium doses of GC in the induction of remission [34]. We infer that the difference in GC dose and tapering did not cause much bias. On the other hand, the differences in usage of IM might be the reason why no risk factors for remission induction failure were identified in the combination therapy group.

## Conclusions

In conclusion, according to our definition, 26 patients (12.1%) failed of remission induction (16 (20.8%) with GC monotherapy, and 10 (7.2%) with combination therapy comprising GC and IM). The lacrimal glands and lungs were the most common sites of remission induction failure. Eosinophilia, higher baseline RI, involvement of more than five organs and dacryoadenitis were risk factors for remission induction failure with GC monotherapy, and combination therapy of GC and IM should be considered for patients with more than three of these risk factors. After the remission-induction stage, patients who failed of remission induction had significantly higher ESR, CRP and IgG4.

## Additional files

Additional file 1: Outcomes of remission induction in the patients diagnosed with definite, probable and possible IgG4-RD. (DOCX 71 kb)

Additional file 2: Side effects observed during treatment. (DOCX 68 kb)

Additional file 3: Clinical features, treatments and outcomes of the patients with persistently active disease. (DOCX 70 kb)

Additional file 4: Clinical features, treatments and outcomes of the patients who relapsed during GC tapering. (DOCX 130 kb)

## Abbreviations

AZA: Azathioprine
CRP: Hyper-sensitivity C-reactive protein
CT: Computed tomography
CTX: Cyclophosphamide
CyA: Cyclosporine A
ESR: Erythrocyte sedimentation rate
GC: Glucocorticoids
IgG4-RD: IgG4-related disease
IM: Immunosuppressive agents
LEF: Leflunomide
MMF: Mycopheolatemofetil
MP: Methylprednisolone
MRI: Magnetic resonance imaging
MTX: Methotrexate
OR: Odds ratio
PET-CT: Positron emission tomography/computed tomography
Ref: Reference
RI: Responder index
T2: Tripterysium glycosides
ULN: Upper limit of normal
