# Supplementary material for: Failure of remission induction by glucocorticoids alone or in combination with immunosuppressive agents in IgG4-related disease: a prospective study of 215 patients
Source: Arthritis Res Ther. 2018 Apr 10;20:65. doi: 10.1186/s13075-018-1567-2 (PMC5894179; doi:10.1186/s13075-018-1567-2)
Supplement: Supplementary file 1 — Outcomes of remission induction in the patients diagnosed with definite, probable and possible IgG4-RD. (DOCX 71 kb) [file 13075_2018_1567_MOESM1_ESM.docx]

**Additional file 1. Outcomes of remission induction in the patients diagnosed with definite, probable and possible IgG4-RD**

| Treatment  Diagnosis | GC monotherapy  Definite Probable Possible P-value^*^  (n=35) (n=2) (n=40) | | | | GC + IM combination therapy  Definite Probable Possible P-value^*^  (n=67) (n=8) (n=63) | | | |
| --- | --- | --- | --- | --- | --- | --- | --- | --- |
| Failure of remission  induction | 8 (22.9%) | 1 (50%) | 7 (17.5%) | 0.577 | 4 (6%) | 0 (0%) | 6 (9.5%) | 0.512 |
| RI reduction <50% | 1 (2.9%) | 0 (0%) | 1 (2.5%) | 1 | 1 (1.5%) | 0 (0%) | 0 (0%) | 1 |
| Relapse | 7 (20%) | 1 (50%) | 6 (15%) | 0.559 | 3 (4.5%) | 0 (0%) | 6 (9.5%) | 0.3 |
| Failure of GC  tapering | 5 (14.3%) | 0 (0%) | 3 (7.5%) | 0.47 | 4 (6%) | 0 (0%) | 5 (7.9%) | 0.732 |

*The patients were divided into definite/probable disease (diagnosed with biopsy) and possible disease (diagnosed without biopsy), and analyzed by Fisher’s exact test.
