# Supplementary material for: Failure of remission induction by glucocorticoids alone or in combination with immunosuppressive agents in IgG4-related disease: a prospective study of 215 patients
Source: Arthritis Res Ther. 2018 Apr 10;20:65. doi: 10.1186/s13075-018-1567-2 (PMC5894179; doi:10.1186/s13075-018-1567-2)
Supplement: Supplementary file 2 — Side effects observed during treatment. (DOCX 68 kb) [file 13075_2018_1567_MOESM2_ESM.docx]

**Additional file 2. Side effects observed during treatment**

|  | GC monotherapy  (N=77) | GC + IM combination therapy  (N=138) | p-value |
| --- | --- | --- | --- |
| Initial 6 months |  |  |  |
| Diabetes mellitus | 6 (7.8%) | 8 (5.8%) | 0.575 |
| Infection | 6 (7.8%) | 13 (9.4%) | 0.805 |
| Liver dysfunction | 0 (0%) | 2 (1.4%) | 0.538 |
| Gastrointestinal  reaction | 3 (3.9%) | 8 (5.8%) | 0.750 |
| All follow-up period |  |  |  |
| Diabetes mellitus | 10 (13%) | 18 (13%) | 1 |
| Infection | 11 (14.3%) | 27 (19.6%) | 0.358 |
| Liver dysfunction | 4 (5.2%) | 3 (2.2%) | 0.252 |
| Leukopenia | 0 (0%) | 3 (2.2%) | 0.554 |
| Gastrointestinal  reaction | 8 (10.4%) | 14 (10.1%) | 1 |
