# Supplementary material for: Failure of remission induction by glucocorticoids alone or in combination with immunosuppressive agents in IgG4-related disease: a prospective study of 215 patients
Source: Arthritis Res Ther. 2018 Apr 10;20:65. doi: 10.1186/s13075-018-1567-2 (PMC5894179; doi:10.1186/s13075-018-1567-2)
Supplement: Supplementary file 3 — Clinical features, treatments and outcomes of the patients with persistently active disease. (DOCX 70 kb) [file 13075_2018_1567_MOESM3_ESM.docx]

**Additional file 3. Clinical features, treatments and outcomes of the patients with persistently active disease**

| Patient no. | Sex/age | Diagnosis | Organ involvement | Treatment for remission induction | Outcome | Follow-up (months) |
| --- | --- | --- | --- | --- | --- | --- |
| 1 | F/28 | Possible | Pancreas + salivary gland + lacrimal gland + lung + lymph node | GC | Persistent lacrimal gland enlargement and pulmonary manifestation, resolved after addition of AZA. | 45 |
| 2 | F/41 | Definite | Bile duct + lacrimal glands + kidney + liver + thyroid gland + lymph nodes | GC | Failure of remission induction. | 6 |
| 3 | M/37 | Definite | Lung + lymph nodes | GC+CTX+T2 | Disease progression despite treatment of GC, CTX, AZA, MMF, FK-506, MP pulse therapy and rituximab. | 39 |

Abbreviations: GC: glucocorticoids; CTX: cyclophosphamide; AZA: azathioprine; T2: tripterysium glycosides; MMF: mycopheolatemofetil; MP: methylprednisolone
