# Supplementary material for: Failure of remission induction by glucocorticoids alone or in combination with immunosuppressive agents in IgG4-related disease: a prospective study of 215 patients
Source: Arthritis Res Ther. 2018 Apr 10;20:65. doi: 10.1186/s13075-018-1567-2 (PMC5894179; doi:10.1186/s13075-018-1567-2)
Supplement: Supplementary file 4 — Clinical features, treatments and outcomes of the patients who relapsed during GC tapering. (DOCX 130 kb) [file 13075_2018_1567_MOESM4_ESM.docx]

**Additional file 4. Clinical features, treatments and outcomes of the patients who relapsed during GC tapering**

| Patient no. | Sex/age | Diagnosis | Treatment for remission induction | Relapse time (month) | Relapsed site | Alternative therapy | Outcome | Follow-up (months) |
| --- | --- | --- | --- | --- | --- | --- | --- | --- |
| 4 | M/70 | Definite | GC | 2 | Lung | IVMP 🡪 GC + CTX | Remission | 21 |
| 5 | M/68 | Possible | GC | 4 | Pancreas + bile duct | GC + MMF | Remission | 12 |
| 6 | M/46 | Definite | GC | 4 | Salivary glands + lymph nodes | GC + CTX | Remission | 30 |
| 7 | M/49 | Definite | GC | 5 | Lacrimal glands + sinus | GC + CTX | Remission | 15 |
| 8 | M/15 | Probable | GC | 6 | Lacrimal glands + lymph nodes + lung | GC + MTX | Remission | 21 |
| 9 | M/50 | Possible | GC | 6 | Lacrimal glands + salivary glands | GC + MTX | Remission; secondary relapse after 4 months | 10 |
| 10 | F/42 | Possible | GC | 3 | Bile duct | GC + AZA | Remission; secondary relapse after 8 months | 33 |
| 11 | M/51 | Definite | GC | 3 | Sinus + lymph nodes | Increased dose of GC + MMF | Remission; secondary relapse after 11 months | 24 |
| 12 | F/54 | Definite | GC | 5 | Lacrimal glands | GC + MMF 🡪 GC + LEF 🡪 GC + CTX | Remission; secondary relapse after 18 months | 23 |
| 13 | M/54 | Possible | GC | 6 | Lacrimal glands + salivary glands + skin | GC + CTX | Remission; secondary relapse after 26 months | 39 |
| 14 | F/61 | Possible | GC | 4 | Liver + lung | GC + CTX | Remission; secondary relapse after 37 months | 45 |
| 15 | M/46 | Definite | GC | 6 | Lymph nodes + lacrimal glands + skin | GC + MTX 🡪 GC + MTX + T2 🡪 GC + CTX 🡪 GC + MMF | Persistently active disease | 29 |
| 16 | M/51 | Definite | GC | 6 | Lung | MP pulse therapy 🡪 GC + CTX | Persistently active disease | 15 |
| 17 | M/44 | Possible | GC | 6 | Lacrimal glands | GC + CTX | Loss of follow-up | 6 |
| 18 | M/64 | Definite | GC + CTX | 2 | Pancreas | IVMP 🡪 GC + MMF | Remission | 23 |
| 19 | M/72 | Possible | GC + CTX | 6 | Pancreas | Increased dose of GC + MMF | Remission | 36 |
| 20 | M/21 | Possible | GC + MTX | 6 | Lacrimal glands | Increased dose of GC + MTX | Remission | 75 |
| 21 | M/54 | Possible | GC + MMF | 6 | Pancreas | Increased dose of GC + MMF + T2 | Remission; secondary relapse after 6 months | 26 |
| 22 | M/48 | Definite | GC + MMF | 6 | Lacrimal glands | GC + CTX | Remission; secondary relapse after 17 months | 23 |
| 23 | M/71 | Possible | GC + MMF | 6 | Pancreas | Increased dose of GC + MMF | Remission; secondary relapse after 19 months | 37 |
| 24 | M/57 | Definite | GC + CTX | 3 | Lacrimal glands + salivary glands + skin | MP pulse therapy 🡪 GC + CTX + AZA 🡪 GC + AZA + LEF 🡪 GC + AZA + T2 🡪 GC + CTX + MTX | Persistently active disease for more than 3 years | 60 |
| 25 | M/60 | Possible | GC + CTX | 6 | Lacrimal glands | Increased dose of GC + T2 🡪 GC + AZA | Difficulty of GC tapering due to recurrent lacrimal gland swelling | 45 |
| 26 | F/47 | Possible | GC + CTX | 6 | Lung + aorta | MP pulse therapy | Loss of follow-up | 6 |

Abbreviations: GC: glucocorticoids; CTX: cyclophosphamide; MMF: mycopheolatemofetil; MTX: methotrexate; AZA: azathioprine; LEF: leflunomide; T2: tripterysium glycosides; MP: methylprednisolone; IVMP: intravenous methylprednisolone
